# Empowering the detection of ChIP-seq “basic peaks” (bPeaks) in small eukaryotic genomes with a web user-interactive interface

**DOI:** 10.1186/s13104-018-3802-y

**Published:** 2018-10-04

**Authors:** Thomas Denecker, Gaëlle Lelandais

**Affiliations:** Institut de Biologie Intégrative de la Cellule (I2BC), Centre National de la Recherche Scientifique: UMR9198, Université Paris-Saclay, Université Paris-Sud - Paris 11, 11 - Bâtiment 400, Orsay, France

**Keywords:** ChIP-seq, Peak calling, Protein DNA-binding sites, Small eukaryotic genomes, bPeaks

## Abstract

**Objective:**

bPeaks is a peak calling program to detect protein DNA-binding sites from ChIPseq data in small eukaryotic genomes. The simplicity of the bPeaks method is well appreciated by users, but its use via an R package is challenging and time-consuming for people without programming skills. In addition, user feedback has highlighted the lack of a convenient way to carefully explore bPeaks result files. In this context, the development of a web user interface represents an important added value for expanding the bPeaks user community.

**Results:**

We developed a new bPeaks application (bPeaks App). The application allows the user to perform all the peak-calling analysis steps with bPeaks in a few mouse clicks via a web browser. We added new features relative to the original R package, particularly the possibility to import personal annotation files to compare the location of the detected peaks with specific genomic elements of interest of the user, in any organism, and a new organization of the result files which are directly manageable via a user-interactive genome browser. This significantly improves the ability of the user to explore all detected basic peaks in detail.

**Electronic supplementary material:**

The online version of this article (10.1186/s13104-018-3802-y) contains supplementary material, which is available to authorized users.

## Introduction

ChIP-seq, i.e. chromatin immunoprecipitation sequencing, is an experimental approach to analyze protein interactions with DNA [1]. Peak detection (also referred as “peak calling”) consists of identifying all the genomic regions in which a significant enrichment of DNA sequences (or reads) in a ChIP sample is observed compared to a control sample. These regions are expected to represent DNA-binding sites for the studied protein [2]. A considerable number of peak calling software packages have been developed (for instance [3, 4], etc.) and choosing the appropriate software, optimized for a specific biological system of interest, is a prerequisite for successful ChIP-seq data interpretation.

In this context, we proposed a methodology to identify “basic Peaks” (bPeaks) in small eukaryotic genomes [5]. The general idea was to take advantage of simpler peak calling for species with small genome sizes (< 20 Mb). The program bPeaks thus performs an exploration of ChIP-seq results at the nucleotide scale. It uses a sliding window, which compares the read distributions between the immunoprecipitation (IP) sample and a control sample. We implemented the bPeaks program with the R language and the associated package is available at the CRAN website [6]. Since its original publication, the bPeaks R package has been downloaded more than 11,360 times (July 2018) and successfully used to identify DNA-binding sites for different proteins in several yeast species [7–10].

Our colleagues, essentially experimental biologists, appreciate the simplicity of the bPeaks methodology. The program uses a combination of only four thresholds to mimic “good peak” properties, as described by investigators who visually inspect ChIP-seq results on a genome browser [5]. However, they highlighted several difficulties. First, working with an R program is a challenging task for people with only limited bioinformatic skills. Initial installation of the necessary software and libraries, importing of the ChIP-seq data in R, and running a bPeaks search can be excessively difficult for simply technical reasons and may thus be an obstacle to the in-depth analysis of the results. Also, bPeaks generates a large number of output files (several dozen), which are all automatically written and stored in a single operating system (OS) folder. These files were meant to be helpful for further investigation (for example, detection of regulatory motifs), but feedback from users brought to our attention the need for their better organization and documentation. Finally, the bPeaks R package only comprises pre-registered annotations of genes for yeast species because our group’s research activities are focused on functional genomics in yeast. This is an important limitation for researchers interested in other organisms.

We developed a new application to overcome these limitations. Referred hereafter as the “bPeaks App” (bPeaks application), it is used via a web browser and makes it possible to perform all the analysis steps required to identify protein DNA-binding sites with ChIP-seq results in a few mouse clicks. We developed new functionalities in the bPeaks App, relative to the original R package, to (i) evaluate the overall quality of the analyzed ChIP-seq data (Lorenz curve and PBC calculation), (ii) facilitate the exploration of output files (with a user-interactive genome browser), and (iii) upload annotation files to compare the location of the detected peaks with particular genomic elements of interest to the user, in any organism. bPeaks App is an open source program available on Github [11]. It has the advantage that it can be deployed locally (on a personal workstation) or on a server. Here, we present the technical solutions that were chosen and explain the main functionalities of the bPeaks App.

## Main text

### Methods

#### General overview

**Fig. 1:**
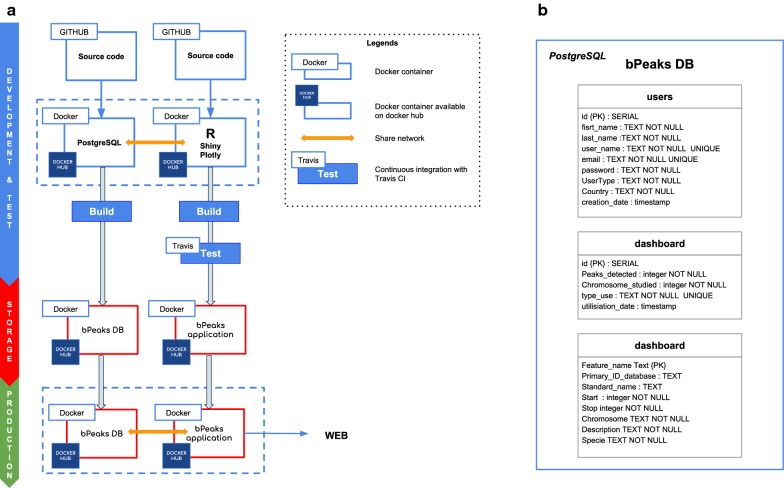
Technical solutions to develop the bPeaks App. **a** Open source softwares used to develop the application are shown here. It is divided into three sections: (1) development and test (in blue), (2) database storage (in red), and (3) final production (in green). **b** Tables defined in the PostgreSQL database that highlight the functioning of the bPeaks App

The bPeaks App is a web application which uses the original bPeaks R package [6]. The backend of the application is based on three mainstream open source technologies: Github [12], Docker [13], and PostgresSQL [14] (see Fig. 1a). The frontend solutions of the application were chosen to provide users a particularly easy-to-use experience using Shiny, the Web Application Framework for R [15]. The Plotly R package [16] was used to obtain dynamic graphical representations, together with Google chart [17]. The application requires a database to control user access. The solution proposed by Shiny requires payment. Thus, we preferred another approach, based on a PostgreSQL database. Put very simply, the database is comprised of three tables: one to manage user information, one to manage an information dashboard and one for gene annotations (Fig. 1b). The connection between R and PostgreSQL was accomplished using RPostgreSQL [18] and the protection of user passwords achieved with the pgcrypto extension.

#### Strategy for versioning the application

Two complementary axes were considered to ensure appropriate versioning of the bPeaks App: (i) R package dependencies and (ii) OS dependencies. The package manager packrat [19] was used to precisely follow the latest versions of all the packages used for development of the bPeaks App. It saves libraries locally and generates a packrat.lock file. This file lists the detailed package versions that were used, including all dependencies. The bPeaks App was built on a containerization paradigm (see Fig. 1a) with Docker, since R software is also dependent on the OS. Our objective was to entirely pack the application and its dependencies in a virtual container. Thus, it was possible to build images that contain everything required for the bPeaks App to function. These images are downloaded on the host system from the Docker Hub ([20, 21]).

#### Installation

The bPeaks App was meant to be installed either on a personal workstation or a laboratory web server. The application can manage several users and multiple simultaneous connections. The bPeaks App can be deployed on Linux, MacOS X, or Windows 10. Detailed information to deploy the bPeaks App can be found in Github README [22]. Minimal requirements are:

- 64 bits OS.
- Docker community edition > v18 (with a minimum of 3 GB of RAM allocated).
- Access to the internet (required for Docker image download).

For deployment on a local workstation, installation scripts are available to create a launcher script to facilitate the use of the bPeaks App. This launcher starts all the components needed to run the application without entering a single command line.

#### Criterion to evaluate ChIP-seq data quality

Lorenz curves and PCR Bottleneck Coefficients (PBCs) are classical criteria to evaluate ChIP-seq data quality [23] and were both implemented into the bPeaks App. Details of the calculations are presented in Additional file 1.

### Results

#### bPeaks App

With the new bPeaks App, our aim was to (1) simplify the use of the bPeaks peak calling method for those with no bioinformatics skills, (2) add several ChIP-seq data representations to assess the overall quality of the initial experiment, and (3) facilitate the exploration of peak calling results. Also, we wanted to guarantee the reproducibility of any results obtained with bPeaks App, systematically tracing all the analysis steps and computational tool versions. We decided to divide the application into two parts referred hereafter as “bPeaks analyzer” and “bPeaks explorer”. bPeaks analyzer focuses solely on the peak calling step. Output files are automatically renamed and reorganized in different OS folders. These files represent the starting point for the bPeaks explorer part, which allows dynamic and user-interactive visualization of the detected peaks, as well as peak localization relative to particular genomic elements (coding or promoter sequences, DNA repeated regions, etc.). All these features in bPeaks explorer are novel compared to previous R package outputs, which were only static files. These sub-applications are accessible after an authentication phase on the home page (see Additional file 2). A study case is shown in Additional file 3 to the illustrate the use of the bPeaks App.

#### bPeaks analyzer to run the detection of peaks

**Fig. 2:**
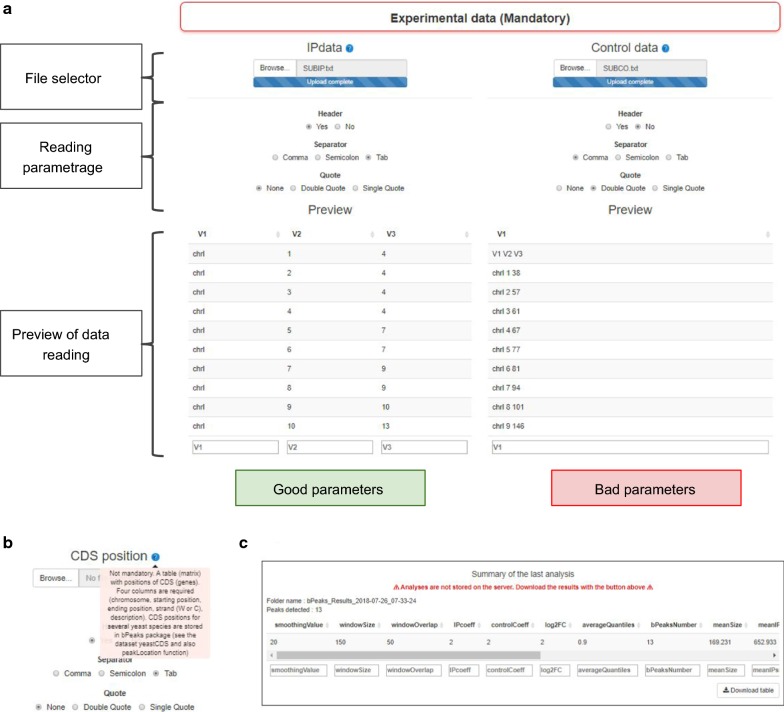
bPeaks analyzer. Screenshots of the web interface associated with the bPeaks analyzer: file selection (**a**), example of help (**b**), and summary of a completed analysis (**c**)

bPeaks analyzer is a web interface to apply the bPeaks method. We paid particular attention to not modify the original R package, so that identical results will be obtained whether bPeaks analyzer is used or not. Figure 2 shows how information required to run bPeaks can be specified in several dedicated areas. Note that help and documentation can be systematically obtained (see Fig. 2b). We maximally simplified the configuration. This is well-illustrated with the functionality of the bPeaks App in reading ChIP-seq data files (IP and control, see Fig. 2a). It is possible to import files with different separators, with the presence or not of a header line, and with the presence or not of quoting characters. Once the selected files are uploaded, a preview is shown, allowing the user to verify that the data importation is correct (see Fig. 2a). Default values for the four thresholds are provided (the same as in the original bPeaks R package). The user can modify the values and run the analysis. Once the analysis is complete, the results are summarized in a table (see Fig. 2c), which can be downloaded. Notably, no file is kept on the server after user sign out. Indeed, during user authentication, a temporary folder is created. All the user analyses and explorations will be saved in this temporary folder. When the user session is over, the folder is deleted. Thus, the bPeaks App does not saturate the workspace memory of the computer on which it is installed. However, the user can download his results at any time (as a zipped file) and save them for future exploration.

#### bPeaks explorer to inspect detected peaks with an interactive genome browser

**Fig. 3:**
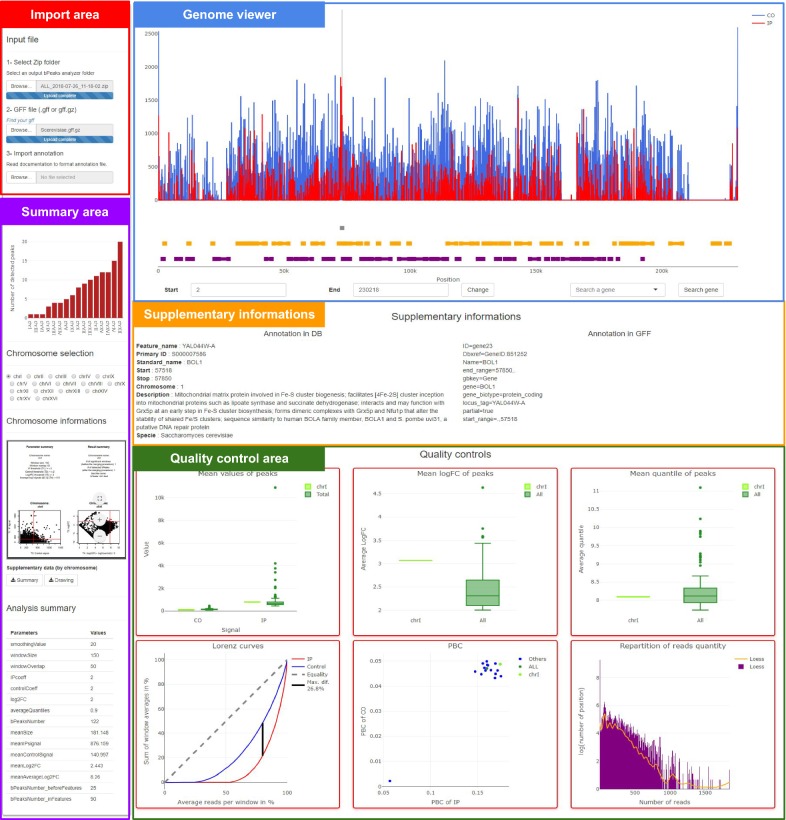
Structure of the bPeaks explorer web page. The page is divided into five parts: import area (red), summary area (purple), genome viewer (blue), supplementary information (orange), and quality control area (green)

bPeaks Explorer is used to generate a graphical and user-interactive overview of the results obtained with bPeaks Analyzer. The web page is divided into five parts (Fig. 3 and more detailed information in Additional file 4):

- An import area, in which the user can upload three files: (1) a zip file generated by the bPeaks Analyzer (see the previous section), (2) an annotation file (GFF format) from the NCBI web service [24] to annotate genomic regions of interest, and (3) a gene annotation file from reference databases (format detailed in Additional file 4).
- A summary area, which is comprised of a table containing a summary of the bPeaks analysis parameters, a barplot showing the number of detected peaks per chromosome, and the graph summary per chromosome.
- A user-interactive genome browser (see below) to explore detected peaks.
- A supplementary information area to obtain information about selected genes or selected peaks.
- A quality control area where six graphs are available (average number of reads detected in the peak, average logFC, average quantile, Lorenz curves, and PBC and repartition of read quantity).

### Discussion

The objective of the bPeaks App is to empower the use of bPeaks, an efficient peak-caller in small eukaryotic genomes. With its docker, there is no need to worry about installing R and the necessary packages. Indeed, all packages and their dependencies are installed in the image available on Docker Hub. We used a package manager to allow reproducibility of the results. Thus, it is possible to reproduce an analysis with the same packages. Through the use of Shiny, the user does not need any computer or programming skills. The user is guided to enter the various parameters. To help him, information bubbles are available at each step. We provide quality controls (Lorenz curve, PBC, etc.) to validate the experimental part. Thus, the user will know whether the analyses are of good quality or not before exploration. Finally, the exploration of results is greatly simplified through the use of Plotly and its dynamic graphics. The user can browse the genome, chromosome by chromosome, and explore the various detected peaks. In conclusion, we propose a completely open source, free, and user-friendly solution for the detection of binding sites between protein and DNA in eukaryotes with small genomes.

## Limitations

The implementation strategies and packages used in R limit the use of bPeaks explorer to organisms with small genomes (< 20 Mb). Moreover, there is no choice in the peak calling strategy. The application only uses and manages results from bPeaks.

## Additional files

**Additional file 1.** Criteria to evaluate ChIP-seq data quality. Illustrated calculation method of the quality criteria: Lorenz curves and PBC.

**Additional file 2.** Connection to the web interface. Authentication details and starting the bPeaks App.

**Additional file 3.** A use case in the yeast *Saccharomyces cerevisiae* with transcription factor Pdr1.

**Additional file 4.** Detailed description of the main parts of the bPeaks explorer application.

## Abbreviations

ChIP-seq: chromatin immunoprecipitation sequencing
CO: control
DNA: deoxyribonucleic acid
IP: immunoprecipitation
OS: operating system
PBC: PCR bottleneck coefficient
