# Supplementary material for: Empowering the detection of ChIP-seq “basic peaks” (bPeaks) in small eukaryotic genomes with a web user-interactive interface
Source: BMC Res Notes. 2018 Oct 4;11:698. doi: 10.1186/s13104-018-3802-y (PMC6172709; doi:10.1186/s13104-018-3802-y)
Supplement: Supplementary file 1 — Additional file 1. Criteria to evaluate ChIP-seq data quality. Illustrated calculation method of the quality criteria: Lorenz curves and PBC. [file 13104_2018_3802_MOESM1_ESM.docx]

### Additional file 1 - Criteria to evaluate ChIP-seq data quality

#### Lorenz curves

Lorenz Curves were initially used in economics [[1]](https://paperpile.com/c/Y0oPKP/Soyn). Their objective was to represent the distribution of income or wealth. In biology, Lorenz Curves are used to find inequalities in distribution. For ChIP-seq data, we can expect to observe a large number of reads in a small number of genomic intervals in an IP sample (thus in precise places forming peaks). It is therefore expected to observe only a few genomic positions with many reads. This characteristic results in a growth curve with a “late” profile. In contrast, we can expect to find a homogeneous distribution of reads along the genome in the control sample, leading to a "constant" growth curve. The Lorenz curves present these two representations and makes it possible to compare the distribution of reads and thus the quality of the immunoprecipitation (Figure 1A of additional file 1). An equality curve is added to check the quality of the control. A control curve close to the equality curve is a guarantee of quality because the read distribution is globally uniform. A large maximum deviation between the two Lorenz curves is also a guarantee of good quality.

#### PBC coefficient

The second quality score is the PBC, a measure of library complexity. A library is composed of all the reads obtained after the sequencing step in ChIP-seq experiments. The complexity of the library is determined by the diversity of the reads that compose it. The PBC has a value between 0 and 1 and is calculated as follows:

$$PBC = \frac{N_{1}}{N_{d}}$$

with

- N_1_: genomic positions with exactly one aligned read
- N_d_ : genomic positions with ≧ 1 aligned reads

Various thresholds are used to interpret the PBC (see Figure 1B of additional file 1 for an illustration):

- 0-0.5 represents severe bottlenecking,
- 0.5-0.8 represents moderate bottlenecking,
- 0.8-0.9 represents mild bottlenecking,
- 0.9-1.0 represents no bottlenecking.

Very low values may indicate a technical problem, such as PCR bias, or a biological finding, such as protein binding sites. The reverse is not good either because it may indicate technical problems with libraries. In our case, a low PBC is desired, because when searching for a binding site, reads accumulate to form peaks, otherwise called bottlenecking in the PBC.


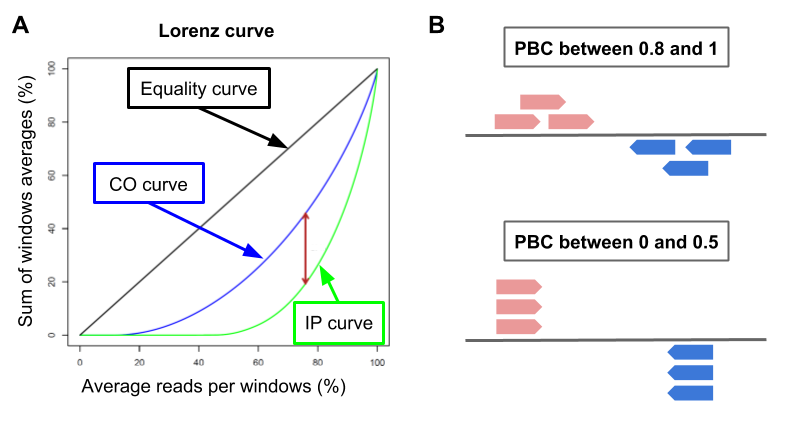


**FIGURE 1 OF ADDITIONAL FILE 1. Criteria to evaluate ChIP-seq data quality in the bPeaks App**. Example of a Lorenz curve (A) and a schematic representation of the PBC according to read alignment (B).

#### Reference of additional file 1

[1. Lorenz MO. Methods of Measuring the Concentration of Wealth. Publications of the American Statistical Association. 1905;9:209.](http://paperpile.com/b/Y0oPKP/Soyn)
