# Supplementary material for: Empowering the detection of ChIP-seq “basic peaks” (bPeaks) in small eukaryotic genomes with a web user-interactive interface
Source: BMC Res Notes. 2018 Oct 4;11:698. doi: 10.1186/s13104-018-3802-y (PMC6172709; doi:10.1186/s13104-018-3802-y)
Supplement: Supplementary file 2 — Additional file 2. Connection to the web interface. Authentication details and starting the bPeaks App. [file 13104_2018_3802_MOESM2_ESM.docx]

## Additional file 2 - Connection to the web interface

After its installation on a personal computer or a shared server (see Methods, main text), the bPeaks App can be used through a web interface (see Figures 2 and 3, main text). This requires users to create an account and sign in to the bPeaks App session they are using (see Figure 1A of additional file 2). Indeed, user authentication was developed to access the bPeaks App (see Figure 1 of additional file 2). This is of particular interest when the application is shared between multiple users, *via* installation on a lab server. The authentication page is divided into four parts :

1. An authentication area: the application queries a PostgreSQL database with the username or email and a password (which is encrypted). In case of error, an alert message is automatically sent, using the shinyalert package [[1]](https://paperpile.com/c/eBVRqf/wFrf)
2. An information area for sign up: a new user can find the procedure to request access to the application.
3. An area to change passwords: once the administrator has accepted a new user, a temporary password is generated and transmitted to the new user. He/she will have to modify their password the first time they connect to the application.
4. An area to add a new user: only the person who is in charge of the bPeaks App administration can do this (a username / password control is performed). After registration, the administrator sends a temporary password to the new user.

After user authentication, a welcome page gives access to either the bPeaks analyzer or the bPeaks explorer (see Figure 1B of additional file 2). A dashboard is also shown on the home page (see Figure 1C of additional file 2) to summarize the application activity, *i.e.* number of connections, number of peaks detected, number of chromosomes parsed for peak calling, etc. A table with the emails of the users who subscribed to the current session of the bPeaks App, is also presented, together with a map with the countries where users are from and two timelines displaying the number of bPeaks analyses or bPeaks explorations which were performed per day.


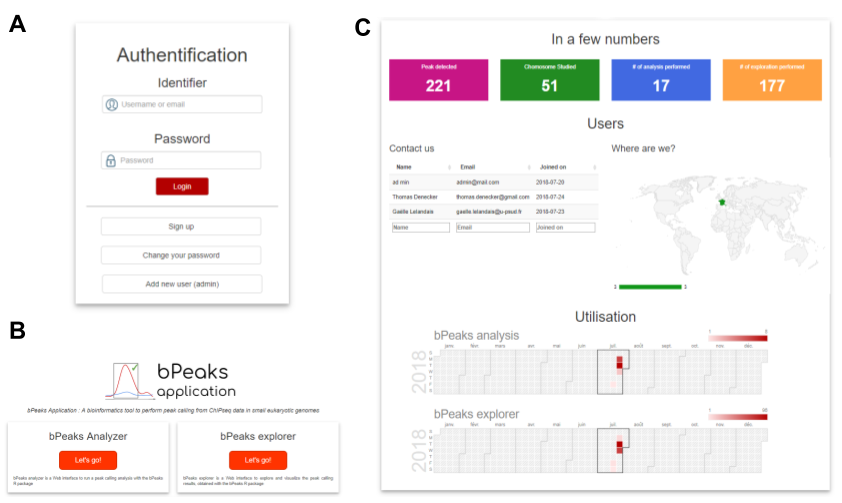


**FIGURE 1 OF ADDITIONAL FILE 2 | Home page of the bPeaks App.** Screenshots are presented: authentication area (A), dual access to the bPeaks analyzer and the bPeaks explorer part (see main text) (B), and the dashboard summarizing the activity of the bPeaks App (see main text)(C).

#### Reference of additional file 2

[1. Attali D, Edwards T. shinyalert: Easily Create Pretty Popup Messages (Modals) in “Shiny.” 2018.](http://paperpile.com/b/eBVRqf/wFrf) [https://CRAN.R-project.org/package=shinyalert](https://cran.r-project.org/package=shinyalert)[.](http://paperpile.com/b/eBVRqf/wFrf)
