# Supplementary material for: Empowering the detection of ChIP-seq “basic peaks” (bPeaks) in small eukaryotic genomes with a web user-interactive interface
Source: BMC Res Notes. 2018 Oct 4;11:698. doi: 10.1186/s13104-018-3802-y (PMC6172709; doi:10.1186/s13104-018-3802-y)
Supplement: Supplementary file 3 — Additional file 3. A use case in the yeast Saccharomyces cerevisiae with transcription factor Pdr1. [file 13104_2018_3802_MOESM3_ESM.docx]

## Additional file 3 - A use case in the yeast *Saccharomyces cerevisiae*

### The data used

Here, we illustrate the use of the bPeaks App by reproducing the case study presented in the bPeaks original article [[1]](https://paperpile.com/c/6CYKFD/eOiq). ChIP-seq experiments were conducted to identify the genomic regions that interact with the Pdr1 transcription factor in the yeast *Saccharomyces cerevisiae*. Pdr1 is a transcription factor that regulates the pleiotropic drug response [[2]](https://paperpile.com/c/6CYKFD/4YeW). The experimental conditions of the data used in this section are presented in [[3]](https://paperpile.com/c/6CYKFD/QsvTC). Two samples were sequenced simultaneously (IP and CO) and peak identification performed using pPeaks App. pPeaks App detected 122 peaks using the default settings (Supplementary Data 2). This number is identical to these presented in the bPeaks paper.

### Exploration of quality scores

First, quality controls are analyzed. The Lorenz curve of the CO sample is relatively close to the equality curve (see Figure 1A of additional file 3- graph on the top). The distribution of the reads is thus globally homogeneous. The Lorenz curve of the IP sample is distant from that of the CO sample (with a maximum difference of 26%). In addition, the curve has a late, bent appearance, which means that a small number of positions have many reads. This observation is confirmed by the distribution of the reads (see Figure 1A of additional file 3 - graph at the bottom). The PBC of chromosome XV is grouped with the that of others (see Figure 1A of additional file 3 - graph in the middle). It is low, but it is common to find low PCBs in transcription factor ChIP-seq experiments. Note that the PBC of the mitochondrial chromosome is completely isolated from the others. There are very few aligned reads. It is therefore not interpretable. In conclusion, these quality controls allow interpretation of the results of the analysis.

### Exploration of transcription factor binding sites

Using the genome viewer, it is very simple to locate the detected peaks (see Figure 1B of additional file 3). The IP signal is shown in red, control signals in blue, gene locations in orange or purple, and genomic positions (peaks) detected with the bPeaks App in gray. To illustrate the use of the pPeaks App, we looked for the PDR5 promoter (described in the reference article). The description presented in annotation DB show that PDR5 is a plasma membrane ATP-binding cassette (ABC) transporter and a multidrug transporter actively regulated by Pdr1 (see Figure 1C of additional file 3). The peak detected is upstream of the gene at the promoter. The presence of the peak is therefore consistent with biology. Finally, supplementary information provides a set of information on this peak of interest, such as its sequence for future explorations (see Figure 1D of additional file 3).


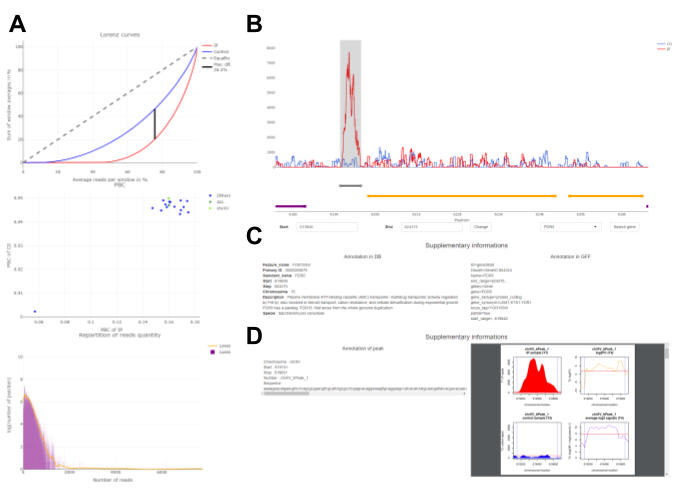


**FIGURE 1 OF ADDITIONAL FILE 3 | ChIP-seq Pdr1 data exploration**. When the quality controls are validated (Lorenz Curves, PBC graph, and the read distribution in that order (A)), it is possible to confidently explore the detected peaks (B). Here, we focus on a peak close to the PDR5 gene in chromosome XV (B). Clicking on the orange segment shows supplementary information about PDR5 (C). Clicking on the gray segment shows supplementary information about this peak (D).

#### References of additional file 3

[1. Merhej J, Frigo A, Le Crom S, Camadro J-M, Devaux F, Lelandais G. bPeaks: a bioinformatics tool to detect transcription factor binding sites from ChIPseq data in yeasts and other organisms with small genomes. Yeast. 2014;31:375–91.](http://paperpile.com/b/6CYKFD/eOiq)

[2. Balzi E, Chen W, Ulaszewski S, Capieaux E, Goffeau A. The multidrug resistance gene PDR1 from Saccharomyces cerevisiae. J Biol Chem. 1987;262:16871–9.](http://paperpile.com/b/6CYKFD/4YeW)

[3. Merhej J, Frigo A, Le Crom S, Camadro J-M, Devaux F, Lelandais G. bPeaks: a bioinformatics tool to detect transcription factor binding sites from ChIPseq data in yeasts and other organisms with small genomes. Yeast. 2014;31:375–91.](http://paperpile.com/b/6CYKFD/QsvTC)
