# Supplementary material for: Empowering the detection of ChIP-seq “basic peaks” (bPeaks) in small eukaryotic genomes with a web user-interactive interface
Source: BMC Res Notes. 2018 Oct 4;11:698. doi: 10.1186/s13104-018-3802-y (PMC6172709; doi:10.1186/s13104-018-3802-y)
Supplement: Supplementary file 4 — Additional file 4. Detailed description of the main parts of the bPeaks explorer application. [file 13104_2018_3802_MOESM4_ESM.docx]

## Additional file 4 – the bPeaks explorer part in details

### Import area

Three files can be uploaded into the import area:

- A zip file generated by the bPeaks Analyzer. An example is available in the Data folder in the Github repository (example.zip) [[1]](https://paperpile.com/c/5r1iih/sjqD).
- An annotation file (GFF format) from the NCBI web service [[2]](https://paperpile.com/c/5r1iih/r2D1d). This file will be used to annotate genomic regions of interest. It is read (compressed or not) by the package ape [[3]](https://paperpile.com/c/5r1iih/GoCz). An example is available in the Data folder in the Github repository (Scerevisiae.gff.gz) [[1]](https://paperpile.com/c/5r1iih/sjqD).
- A gene annotation file from reference databases. This file is composed of nine columns in tsv format: a feature name (mandatory), a primary database ID, a standard name, a start position, a stop position, a chromosome name, a description field, and a species name. An example is available in the Database folder in the Github repository (Annotation_SCERE.tsv) [[4]](https://paperpile.com/c/5r1iih/xKw6).

### User interactive genome viewer

The genome viewer offers a dynamic exploration of the results (Figure 3, main text - blue area). The graph is generated using Plotly, which offers highly useful tools (snapshot, selection, scale, etc. see Methods). The graph is composed of two lines (one for the IP sample and one for the control sample) and vertical gray bars corresponding to detected peaks. Below, three types of colored segments can be displayed, corresponding to gene and peak annotations. If a GFF file has been imported, two of these segments are colored in pink for the Watson DNA strand (+) and purple for the Crick DNA strand (-). By hovering over these segments, the information available in the GFF is displayed (Figure 1B of additional file 3). The last bar in gray corresponds to the peak annotations. By hovering over this segment, information about the peak is displayed, such as, for example, its sequence. Also, to display a specific region of a chromosome, the user can define a specific region, specifying the beginning and the end positions. The graph will then be automatically reloaded with these coordinates as the limit. Finally, if a GFF file is imported, the user can search for a specific gene name. To help him, only the genes detected in GFF file are in the search area and the possibilities are reduced according to what is entered.

### A supplementary information area

In this area, two types of information can be displayed (Figure 3, main text - orange area). The first type of information consists of gene annotation. When the user imports a GFF file, colored bars appear in the genome viewer, corresponding to the genes. Each gene has an annotation in the GFF file (sequence, source, feature, start, end, score, strand, phase, and attributes (see [[5]](https://paperpile.com/c/5r1iih/79kHH), where the format is described in Ensembl)). As there is no gene description in GFF files, we added a table in the bPeaks App database (see Annotation, Figure 1B, main text). This table stores the important information available in the reference databases (Saccharomyces Genome Database (SGD, [[6]](https://paperpile.com/c/5r1iih/8Aq9p)), Candida Genome Database (CGD, [[7]](https://paperpile.com/c/5r1iih/mxviH)),...). To import new data into this table (for other species than yeasts), an import button is available in the import area. When importing data, the application will check whether the table is in the right format and then it will check the data line by line. If there is an error during the import process, a help message appears to help the user to understand the origin of the problem and the row of the concerned table. A common error is the presence of a simple quote (also used by PostgreSQL). It is also possible to update the already present data using the same approach. If the gene is already present in the database, the new data will be associated with it. To display the annotation of a gene, one simply clicks the mouse on one extremity of the gene. Two types of annotations will be provided: (1) the annotation from the reference databases, if available in the database, and (2) the annotation from the GFF file.

The second set of information available in this area is that of the peak. When the user clicks on a grey segment, annotations of the selected peak and graphical representations are displayed. For each position in the peak, four representations are proposed: the IP sample values, the control sample values, the log2FC values, and the average log2FC signals. These representations allow assessment of the biological relevance of the genomic regions identified with the bPeaks analyzer. Information about peak includes, for example, the start and end positions of the peak, its name, and its sequence. To have the sequence of a peak, it is necessary, during the analysis, to import a fasta file containing the sequence of the reference genome (the same file used during the read alignment, in ChIP-seq data pre-processing). This information for all detected peaks is available in the same file that can be used with other tools to deeply analyze the identified genomic regions.

### Quality control area

This area is composed of six graphs (Figure 3, main text - green area). The first three are boxplots: average number of reads in detected peaks, average logFC, and average quantile. Their objective is to observe whether there is a difference in the distribution along the chromosome in relation to the whole. The next graph consists of the Lorenz representation (see Methods). It is composed of IP and CO Lorenz curves (blue and red, respectively) and an equality curve (gray). The black curve corresponds to the maximum difference between the two curves (of which the value is written on the graph). The following graph shows the PBC of the control sample as a function of the PBC of the IP sample (see Methods for PBC definition). This representation makes it possible to rapidly determine whether a chromosome is different from the others (Figure 3, main text - for example, the mitochondrial chromosome in bottom left). Finally, the last graph shows the number of positions (log transformed) with a given number of reads. Normally, there should be few positions with many reads. A Loess curve has been added to see the trend (yellow curve). These graphs all allow the user to evaluate the relevance of the exploration.

#### References of additional file 4

[1. Data folder with examples to use bPeaks App. Github.](http://paperpile.com/b/5r1iih/sjqD) <https://github.com/thomasdenecker/bPeaks-application/blob/master/Data/.> [Accessed 2 Aug 2018.](http://paperpile.com/b/5r1iih/sjqD)

[2. NCBI - Genome. NCBI.](http://paperpile.com/b/5r1iih/r2D1d) <https://www.ncbi.nlm.nih.gov/genome.> [Accessed 31 Jul 2018.](http://paperpile.com/b/5r1iih/r2D1d)

[3. Paradis E, Claude J, Strimmer K. APE : analyses of phylogenetics and evolution in R language. Bioinformatics. 2004;20:289–90.](http://paperpile.com/b/5r1iih/GoCz)

[4. Database folder with an annotation example. Github.](http://paperpile.com/b/5r1iih/xKw6) <https://github.com/thomasdenecker/bPeaks-application/tree/master/Database.> [Accessed 2 Aug 2018.](http://paperpile.com/b/5r1iih/xKw6)

[5. Ensembl - GFF. Ensembl.](http://paperpile.com/b/5r1iih/79kHH) <https://www.ensembl.org/info/website/upload/gff.html.> [Accessed 31 Jul 2018.](http://paperpile.com/b/5r1iih/79kHH)

[6. Cherry JM, Hong EL, Amundsen C, Balakrishnan R, Binkley G, Chan ET, et al. Saccharomyces Genome Database: the genomics resource of budding yeast. Nucleic Acids Res. 2012;40 Database issue:D700–5.](http://paperpile.com/b/5r1iih/8Aq9p)

[7. Skrzypek MS, Binkley J, Binkley G, Miyasato SR, Simison M, Sherlock G. The Candida Genome Database (CGD): incorporation of Assembly 22, systematic identifiers and visualization of high throughput sequencing data. Nucleic Acids Res. 2017;45:D592–6.](http://paperpile.com/b/5r1iih/mxviH)
